# Aortic Rupture during Endovascular Aneurysm Repair. Report of Our Experience and Review of the Literature

**DOI:** 10.1055/s-0040-1714123

**Published:** 2020-12-11

**Authors:** Konstantinos G. Moulakakis, Andreas M. Lazaris, John D. Kakisis, George S. Sfyroeras, George Theocharopoulos, Andreas I. Panagiotopoulos, Nikolas Krinos, George Geroulakos

**Affiliations:** 1 Department of Vascular Surgery, Athens University Medical School, Attikon University Hospital, Athens, Greece

**Keywords:** EVAR, intraoperative, aortic rupture, open conversion, acute conversion

## Abstract

**Background** Aortic neck wall rupture during endovascular repair of abdominal aortic aneurysms (EVAR) is an underreported potentially fatal complication. Only a few cases have been reported. The main cause of this complication is repeated attempts at balloon inflation or overdilation to treat an intraoperative Type 1a endoleak. We report three cases complicated by procedure-related aortic neck wall rupture during EVAR. We also review the literature regarding the causes and outcomes of this complication.

**Methods** Medical records of all patients undergoing EVAR between January 2009 and March 2019 were retrospectively reviewed.

**Results** Overall, 824 EVAR procedures were performed, and rupture of the aortic neck wall was observed in three patients. In all cases, a Type 1a endoleak was observed and, in all cases, repeated ballooning attempts had been performed to resolve the endoleaks. In all cases, conversion to open repair was performed and all patients survived.

**Conclusion** In cases of Type 1a endoleak, a maximum of two ballooning attempts should be performed even if a Type 1a endoleak persists. In case of intraoperative aortic neck wall rupture, control of the hemorrhage should be achieved immediately by advancing the balloon above the site of rupture. Emergency surgical conversion in case of hemodynamic stability is the first choice. According to the literature, emergency surgical conversion, especially in cases of endograft with suprarenal fixation, is associated with significant morbidity and mortality rates, mainly due to hemorrhage and to the length of the procedure required to repair the aortic neck wall injury.

## Introduction

Endoleaks are considered significant adverse events after endovascular repair of abdominal aortic aneurysms (EVAR), since persistence of blood flow and pressure in the aneurysm sac may lead to graft failure with an unfavorable outcome. A Type 1a endoleak is defined as a persistent perigraft channel of blood flow caused by a failure of the graft to adequately seal at the proximal landing zone. Type-1a endoleaks are present on the initial angiogram after endograft deployment in 6 to 8% of procedures. There may be excessive graft undersizing or oversizing due to the presence of mural thrombus, angulation, calcification, or neck tapering. 1

Initial management of Type 1a endoleak is angioplasty with a compliant balloon. 1 2 However, repeated attempts at balloon inflation or overdilation can produce high-radial pressures and lead to aortic rupture. Aortic neck rupture during EVAR is an underreported serious emergency situation requiring conversion to open repair in the majority of cases. Rupture is associated with higher morbidity and mortality rates compared with standard open aneurysm repair. 3 4

There are few reports regarding aortic rupture caused by a molding balloon during EVAR. We report three cases complicated by procedure-related aortic neck wall rupture caused by the molding balloon during EVAR. We also review the literature regarding the causes and outcomes of this potentially fatal complication.

## Materials and Methods

A retrospective review of all patients treated with EVAR for abdominal aortic aneurysm (AAA) with commercially available endografts at Attikon General Hospital, Greece, between January 2009 and March 2019 was performed. A team of vascular surgeons performed all EVARs in an operating room equipped with a portable C-arm with angiographic and road-mapping capabilities. Overall, 824 EVAR procedures were performed, and rupture of the aortic wall was observed in three patients. This study was approved by the Committee on Research Ethics at Attikon University Hospital, Athens, Greece.

## Results

### Case 1

**Fig. 1:**
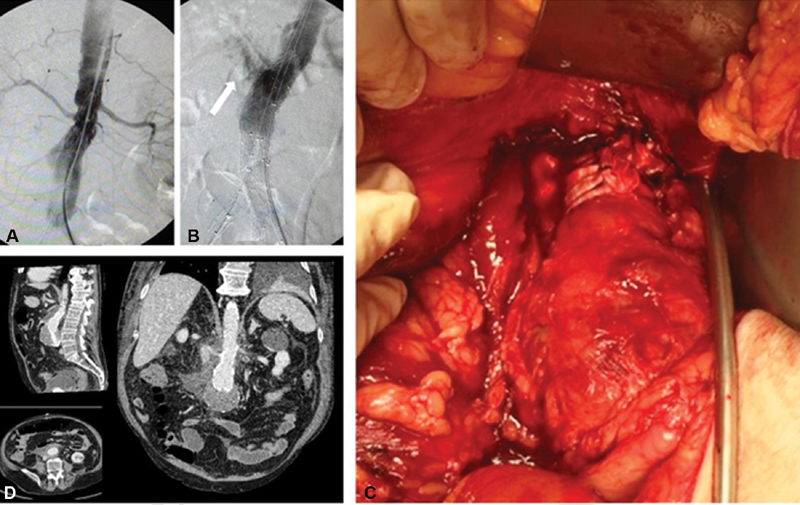
( **A** ) Angiography shows the Type 1a endoleak and ( **B** ) extravasation around the right part of the infrarenal neck of the aorta (white arrow) after balloon inflation. ( **C** ) A conversion to open repair was performed and effective external banding of the infrarenal neck with two polyester limbs, tied in the same fashion, close to one another and parallel just below the renal arteries was performed, also sealing the tear of the aortic wall and stopping the hemorrhage. ( **D** ) A computed tomography angiography on the fifth postoperative day showed complete sealing in the proximal neck with no evidence of endoleak.

An 84-year-old man underwent elective endovascular AAA repair EVAR for a 5.8-cm infrarenal AAA ( Fig. 1A ). 5 The patient's past medical history included chronic pulmonary obstructive disease and arterial hypertension. During the EVAR procedure, a stent-graft (28 mm Zenith, Cook, Inc., Bloomington, IN) was deployed below the lowest right renal artery. The neck aneurysm was conical, with diameters of 22 mm just below the lowest right renal artery and 25-mm, 1-cm distally. Low-pressure ballooning was performed with a Coda 32-mm balloon catheter (Cook, Inc., Bloomington, IN) in the area of the most proximal covered stent and after complete deflation in the distal fixation sites, as well as in the overlap zones between the main body and the iliac limbs. The final angiography revealed a Type 1a endoleak that was attributed to the conical shape and the slight angulation of the infrarenal neck, leading to imperfect apposition of the endograft. Low-pressure reballooning was performed proximally in the neck, but the angiography showed persistence of the Type 1a endoleak. Reballooning with gradual slow inflation was performed for the third time, and we observed a sudden abrupt increase of the diameter of the endograft during balloon inflation. A volume of less than 25 cc was inflated during each ballooning. In the subsequent final angiography, extravasation around the right part of the infrarenal neck of the aorta was observed ( Fig. 1B , white arrow), associated with a sudden and significant decrease in systemic arterial pressure (from 120 to 55 mm Hg). The balloon was immediately advanced and inflated above the renal arteries. Aterial pressure increased, and the patient became hemodynamically stable. Conversion to open repair was performed.

After laparotomy, a large hematoma with active bleeding was found in the retroperitoneal space. The left renal vein was ligated and proximal control was achieved by placement of a vascular clamp above the renal arteries. A longitudinal tear of 0.8-cm length below the right renal artery was found. Initially, we attempted to suture the lesion with 4–0 Prolene stitches. We included the stent graft in the sutures to reinforce the aortic wall, but when we released the clamp, due to the radial tension of the endograft, the lesion was fragile and started bleeding again. We placed again the clamp above the renal arteries. To avoid endograft explantation, control the bleeding, and solve the problem of the Type 1a endoleak, we performed external banding of the infrarenal neck with a polyester prosthesis covering the tear of the aortic rupture and effectively plicating the aortic neck ( Fig. 1C ). 5 The patient's computed angiography 1 week later showed no endoleak ( Fig. 1D ), and the patient was discharged on the sixth postoperative day in good general condition. Three months after the operation, he remains in excellent condition.

### Case 2

A 73-year-old man underwent elective EVAR for a 5.5-cm infrarenal AAA. His past medical history included coronary artery disease, hyperlipidemia, and arterial hypertension. The aneurysm had a conical neck measuring 18 mm in diameter at the level of the renal arteries, 22 mm 1 cm below the arteries, and 24 mm 1.5 cm below the renal areteries. Calcification covering approximately 25% of the aortic neck perimeter was also noted. The advancement of wires and catheters was easy, and a 28-mm Anaconda stent-graft was deployed just below the renal arteries.

**Fig. 2:**
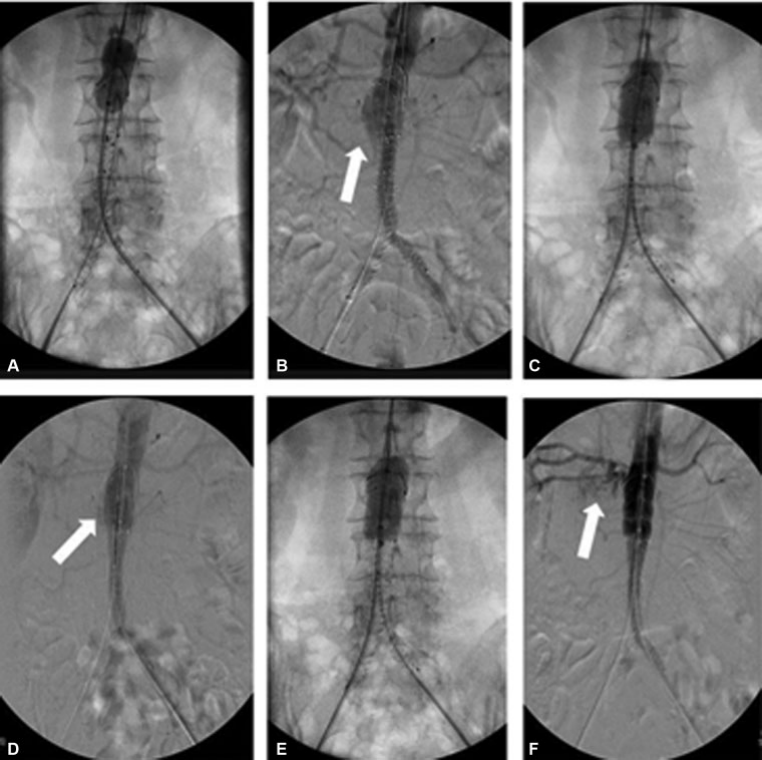
( **A** ) After balloon inflation, ( **B** ) the angiography revealed a Type 1a endoleak (white arrow). ( **C** ) Reballooning was performed proximally in the neck of the aneurysm, ( **D** ) but a new intraoperative angiography revealed the persistency of the Type 1a endoleak, which was reduced (white arrow). ( **E** ) Reballooning was performed once more with less than 25-cc inflation volume. ( **F** ) In the subsequent final angiography, extravasation around the right part of the infrarenal neck of the aorta was observed.

Low-pressure ballooning ( Fig. 2A ) was performed with a Coda 32-mm balloon catheter (Cook, Inc., Bloomington, IN) in the area of the most proximal covered stent, in the distal fixation sites, as well as in the overlap zones between the main body and the iliac limbs. The final angiography revealed a Type 1a endoleak, which was attributed to the conical neck and the presence of the atherosclerotic plaque ( Fig. 2B , white arrow). Reballooning was performed proximally at the neck of the aneurysm ( Fig. 2C ), but a new intraoperative angiography revealed persistence of the Type 1a endoleak, which was reduced ( Fig. 2D , white arrow). Reballooning was performed once more with less than 25-cc inflation volume ( Fig. 2E ). In the subsequent final angiography, extravasation to the right of the infrarenal neck of the aorta was observed. This was associated with a modest decrease of arterial pressure ( Fig. 2F ). Conversion to open repair was performed. A hematoma around the aorta without active bleeding was found. A proximal clamp was placed just below the renal arteries, the sac was excised, and the graft was removed. A tubular 18-mm graft was placed, and the postoperative course was uneventful. The patient was discharged 7 days later in good general condition.

### Case 3

**Fig. 3:**
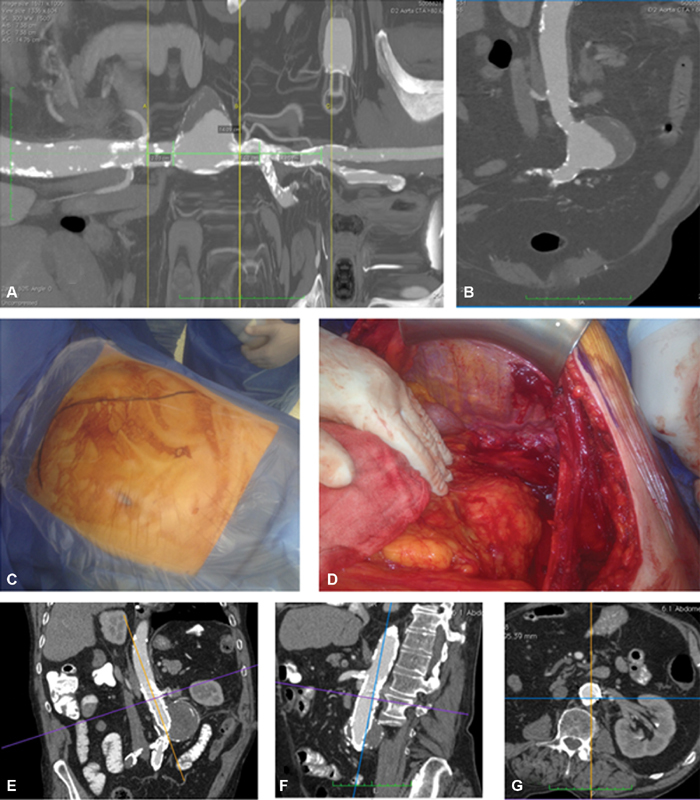
( **A,** **B** ) Regarding the characteristics of the abdominal aortic aneurysm, there was calcification in the proximal neck and the aorta at the level of the bifurcation was 16 mm in diameter and calcified. ( **C,** **D** ) Through a rooftop approach with a left-side visceral rotation, a local hematoma was found with no active hemorrhage, biological glue was applied. ( **E–G** ) He was discharged on the 19th postoperative day in good general condition. At 1-year follow-up, a computed tomography scan showed no evidence of endoleak and complete exclusion of the aneurysm.

A 77-year-old man underwent elective EVAR for a 6.2-cm infrarenal saccular AAA. His past medical history included chronic obstructive pulmonary disease, arterial hypertension, and hostile abdomen due to previous abdominal operations (peritonitis because of small bowel perforation, gastrectomy, and large incisional hernia). Regarding the characteristics of the AAA, there was calcification in the proximal neck (diameter of the neck 19 mm and length 3 cm), and the aorta at the level of the bifurcation was 16 mm in diameter and calcified ( Fig. 3A, B ).

An aortouniliac endograft 22 mm in diameter (Cook, Bloomington, IN) was deployed 1 cm below the left renal artery, intending to avoid the calcified plaque, and sealed in the left common iliac artery, while an occluder plug was inserted into the right common iliac artery. Low-pressure ballooning was performed manually with a Coda 32-mm balloon catheter (Cook, Inc., Bloomington, IN) in the area of the most proximal covered stent and the distal fixation sites, as well as in the overlap zones between the main body and the iliac limbs. The final angiography revealed a Type 1a endoleak, which was attributed to calcification of the infrarenal neck leading to poor apposition of the endograft. Multiple attempts to balloon the proximal neck were performed (with inflation volumes of less than 25 cc), and the patient developed a drop in blood pressure. In the intraoperative angiography, an “ulceration bulge” of the aortic neck was observed without extravasation. A cuff was placed just below the lowest left renal artery covering the bulging area of the aorta. The patient was hemodynamically stable and the procedure was completed with a femoral-femoral bypass.

Two hours later, while the patient was in the recovery room, he developed tachycardia, emergency CT angiography was performed, which revealed a local contained extravasation around the proximal neck of the aorta. The patient was transferred to the operating room; through a rooftop approach with a left-side visceral rotation the proximal aortic neck was exposed. A local hematoma was found with no active hemorrhage ( Fig. 3C, D ). Biological glue was applied, and the abdomen was closed. In the postoperative course, the patient developed atrial fibrillation and depressed pulmonary function. He was discharged on postoperative day 19 in good general condition. At 1-year follow-up, a CT scan showed no evidence of endoleak and complete exclusion of the aneurysm ( Fig. 3E–G ).

## Discussion

Most often, vascular injury during EVAR occurs in the iliac vessels due to severe tortuosity, diminished diameter, and/or calcification of the iliac arteries. 6 Fernandez et al 6 reported a 2.98% chance of iliac rupture during EVAR and 8.9% during TEVAR. 3 More than 95% of these iliac injuries were adequately treated endovascularly with covered stents or limbs.

Aortic neck wall rupture during EVAR is an underreported potentially fatal complication, and only a few cases have been described in the literature. Schlösser et al 7 reviewed the literature regarding early or late aneurysm rupture after EVAR. Overall, 270 patients with ruptures were identified. In 13 cases, the ruptures occurred intraoperatively during endovascular repair, and the mortality rate was 44.4%. Balloon dilatation of proximal aortic neck was the cause of rupture in 4 out of the 13 cases. Other causes were instrumentation and perforation of the aneurysm sac, rupture during attempts to remove insufficiently deployed proximal stent grafts, and poststent deployment. Lee et al 8 reported a case of aortic neck rupture after repeat ballooning for a Type 1a endoleak. The authors explained the rupture as the result of rapid inflation of the balloon catheter. They concluded that gentle, slow inflation of the balloon is important to prevent vascular rupture. Jimenez et al 9 reported two cases of aortic rupture during elective EVAR with a 50% mortality rate, while Verzini et al 10 reported a single-fatal case of aortic rupture that occurred at the bottom of a conical angulated neck during deployment of the endograft.

Our experience with these three cases raises important points that should be highlighted and analyzed as follows:

1. In all cases, a Type 1a endoleak was observed, and in all cases, repeated ballooning attempts were performed to resolve the endoleaks. We believe that if a Type 1a endoleak persists after a maximum two ballooning attempts, other endovascular options should be considered, such as placement of an aortic cuff or use of a Palmaz's stent. 11 If these options also fail to seal the endoleak, coil and/or glue embolization or endostaples can be used at a second stage. 1 12 Chimney grafts and fenestrated cuffs have also been used to treat Type 1a endoleaks.
2. It is well known that an abdominal aortic aneurysm with conical or heavily calcified aortic neck may inhibit good fixation and promote migration and early/late Type 1a endoleak. A conical neck in hostile anatomies represents the strongest factor associated with proximal failure of standard EVAR. 13
3. In all cases, a Coda balloon catheter was used. The Coda is a semicompliant polyurethane balloon used for expansion of AAA endografts. The catheter balloon maintains a constant pressure while conforming to the shape of the endograft in which it is expanded, resulting in greater contact of the balloon with the inner surface of the endograft. According to the instructions for use, overdilatation can lead to aortic rupture. The molding balloon should only be applied within the covered part of the stent graft. The maximum inflation volume is 30 mL for the 32-mm catheter and 40 mL for the 40-mm catheter. We believe that repeated attempts of ballooning in challenging necks, even with less inflation volume than indicated by the user's manual, can lead to aortic neck rupture. Thus, manual slow inflation of the balloon for a maximum of two times is recommended to prevent vascular rupture.
4. Aortic rupture during EVAR is a devastating complication. Prompt diagnosis and treatment can improve the survival of patients who develop this complication. In our experience, all patients survived due to prompt open conversion. According to the literature, mortality from this complication can be high. In the review paper by Schlösser et al, 7 the mortality rate in cases of aortic rupture was 44.4%. Control of the hemorrhage by advancing the balloon above the site of rupture is crucial for the patient to achieve hemodynamic stability.
5. Neck banding is a useful technique for simultaneously treating a Type 1a endoleak and aortic neck rupture during EVAR. 5 13 Effective external banding of the infrarenal neck with polyester prosthesis strips at the level of the rupture may lead to control of the hemorrhage and exclusion of blood flow to the aneurysm sac. 5 14

## Conclusion

During EVAR, a maximum of two ballooning attempts should be performed even if a Type 1a endoleak persists. In cases of aortic neck wall rupture, control of the hemorrhage should be achieved immediately by advancing the balloon above the site of rupture. Emergency surgical conversion in case of hemodynamic stability is the first choice. However, it should be highlighted that emergency surgical conversion, especially in cases of endograft with suprarenal fixation, is associated with significant morbidity and mortality rates, mainly due to hemorrhage and to the length of the procedure required to restore the aortic neck wall injury.
